# A novel methodological approach to participant engagement and policy relevance for community-based primary medical care research during the COVID-19 pandemic in Australia and New Zealand

**DOI:** 10.1186/s12961-023-01100-8

**Published:** 2024-01-22

**Authors:** Katelyn Barnes, Sally Hall Dykgraaf, Kathleen O’Brien, Kirsty Douglas, Kyle Eggleton, Nam Bui, Sabrina T. Wong, Rebecca S. Etz, Felicity Goodyear-Smith

**Affiliations:** 1 Academic Unit of General Practice, ACT Health Directorate, Canberra, ACT Australia; 2 Rural Clinical School, School of Medicine and Psychology, the Australian National University, Canberra, ACT Australia; 3 Present Address: Academic Unit of General Practice, School of Medicine and Psychology, the Australian National University, Canberra, ACT Australia; 4 Department of General Practice & Primary Health Care, University of Auckland, Auckland, New Zealand; 5 School of Nursing and Centre for Health Services and Policy Research, University of British Columbia, 2211 Westbrook Mall, Vancouver, BC V6T2B5 Canada; 6 Larry A. Green Center for the Advancement of Primary Health Care for the Public Good, Department of Family Medicine and Population Health, Virginia Commonwealth University, Richmond, VA United States of America

**Keywords:** COVID-19, Primary care, General practice, Methodology

## Abstract

Community-based primary care, such as general practice (GP) or urgent care, serves as the primary point of access to healthcare for most Australians and New Zealanders. Coronavirus disease 2019 (COVID-19) has created significant and ongoing disruptions to primary care. Traditional research methods have contributed to gaps in understanding the experiences of primary care workers during the pandemic. This paper describes a novel research design and method that intended to capture the evolving impact of the COVID-19 pandemic on primary care workers in Australia and New Zealand. Recurrent, rapid cycle surveys were fielded from May 2020 through December 2021 in Australia, and May 2020 through February 2021 in New Zealand. Rapid survey development, fielding, triangulated analysis and dissemination of results allowed close to real-time communication of relevant issues among general practice workers, researchers and policy-makers. A conceptual model is presented to support longitudinal analysis of primary care worker experiences during the COVID-19 pandemic in Australia and New Zealand, and key learnings from applying this novel method are discussed. This paper will assist future research teams in development and execution of policy-relevant research in times of change and may inform further areas of interest for COVID-19 research in primary care.

**Supplementary Information:**

The online version contains supplementary material available at 10.1186/s12961-023-01100-8.

## Introduction

Community-based primary care, such as general practice or urgent care, serves as the first point of access to healthcare for most Australians and New Zealanders [1–3]. As medical generalists, general practitioners, primary care nurse practitioners and practice nurses provide care for all ages, cultures, injuries and diseases. Being community-based provides opportunity for ongoing and longitudinal care across the lifespan [2, 3]. General practice was recognized as a vital component of health services during previous viral outbreaks, such as the 2009 H1N1 pandemic [4, 5]. The COVID-19 pandemic has seen acknowledgement and involvement of community-based primary care, in particular general practice.

Early in the COVID-19 pandemic, Australia and New Zealand implemented strategies intended to support COVID-19 management and maintain usual medical practice in primary care [6]. Both countries saw rapid and widespread adoption of telehealth and phone triaging for general practice, creation of community-based COVID-19 testing and treatment centres and implementation of electronic prescribing to reduce interactions between healthy and potentially infectious people [6–8]. Personal protective equipment (PPE) for primary care workers was recommended, although access to it was often poor [9–11]. Physical distancing was enforced, with clinics requiring patients to wait outside until being called in for an appointment [11–13]. All strategies required clinics to rapidly create and communicate workplace policies to ensure safety of staff and patients. However, information and policies governing general practice have, and continue to, evolve with volatile COVID-19 contexts [9, 10]. Despite both Australia and New Zealand having an up-to-date pandemic plan, and experiencing fewer COVID-19 infections per capita than other countries between 2020 and 2022 (prior to the omicron variant outbreaks) [14], COVID-19 created significant and ongoing disruptions to primary care. It is important to track, evaluate and report the impact of disruptions in primary care to learn from and adapt future pandemic planning.

Conducting rigorous, peer-reviewed research that can reliably inform health system decision-making has been challenging during the COVID-19 pandemic [15]. Early in the pandemic, evidence used to inform policy relied on lessons learned from non-COVID-19 illnesses (e.g. influenza), which while similar, were not reflective of the global burden and uncertainty related to COVID-19 [5, 6]. Traditional methods to gather experiential evidence during COVID-19 have been slow and of questionable quality [15], with high burden on busy clinicians [16], particularly given the lack of Australian or New Zealand primary care research infrastructure [17, 18]. As such, there was a need for data that could provide immediate utility or benefit, reflective of national or local pandemic contexts, while not placing undue response burden on participants.

Rapid, responsive, repeated, ‘light-touch’ methods have been valuable tools to capture the experiences of primary care workers during the COVID-19 pandemic, and the way that experiences vary across place and time. The objective of the paper is to describe and reflect on a novel, recurrent, rapid-cycle survey method employed to capture the evolving impact of the COVID-19 pandemic on primary care in Australia and New Zealand. The method is based on a similar survey initially developed and fielded in the United States and Canada, beginning in March 2020 [19, 20], and forms part of an international collaborative project among the four countries. Understanding the benefits, drawbacks and potential solutions of this approach may inform or facilitate more responsive and impactful research in future times of significant and precipitous change, uncertainty or crisis.

## Methods

This study used a pragmatic approach [21, 22] and employed responsive, iterative, cross-sectional surveys using a combination of open and closed questions to explore and report the experience of Australian and New Zealand community-based primary care workers over the initial course of the pandemic. The study was conducted in accordance with the Declaration of Helsinki [23] and approved by Australian National University Human Research Ethics Committee (2020/273) and the University of Auckland Human Participants Ethics Committee (024659). The research team included clinicians, researchers, educators and policy-linked academics.

### Setting

Surveys were conducted online, aimed at community-based primary care practitioners in Australia and New Zealand. Surveys were released from May 2020 to December 2021 in Australia, and from May 2020 to February 2021 in New Zealand. Surveys were fielded for 1 week, every 2–4 weeks for 5 months (see Table 1), covering the initial peak of the pandemic, then declined in frequency as the pandemic progressed at various intervals. Results reported are based on available data by date of publication.

**Table 1:** Timing of data collection and flash question themes

Dates fielded	Series Fielded		Flash question theme
	Australia	New Zealand	
22–29 May 2020	1	1	Changes to delivery of care as a result of COVID-19
5–12 June 2020	2	2	Support and practice model changes as a result of COVID-19
19–25 June 2020	3	3	Barriers and enablers to safe and effective telehealth
10–17 July 2020	4	–	Safety from COVID-19 and use of video versus telephone
21–30 July 20	5	4	Mental health presentations
7–13 Aug 2020	6	–	Respiratory presentations to community-based primary care and relationships with COVID-19-specific centres
21–27 Aug 2020	7	5	Respiratory symptom assessment and management (Aus) Respiratory presentations to community-based primary care and COVID-19 impacts on vulnerable populations (NZ)
3–11 Sept 2020	8	6	Experiences for vulnerable patients in general practice
17–24 Sept 2020	9	7	Consequences of delayed care
14–22 Oct 2020	10	8	Barriers to telehealth use
11–19 Nov 2020	11	9	Return to face-to-face care
9–17 Dec 2020	12	10	Resources required to support ongoing telehealth
11–18 Feb 2021	13	11	Vaccination rollout logistics and education needs
18–24 April 2021	14	–	Respiratory symptom assessment and management
9–16 July 2021	15	–	Vaccine counselling in general practice
10–17 Sept 2021	16	–	Community-based primary care clinician involvement in clinical decision-making for their patients positive for COVID-19
9–16 Dec 2021	17	–	Management of patients positive for COVID-19 in the community

### Participant eligibility

Eligible participants were primary care doctors, practice nurses, nurse practitioners or practice managers working in Australian or New Zealand community-based primary care practices from May 2020. Participants were required to work at the same practice for at least 12 months. GP registrars and students were excluded due to the lack of continuity during the study period. Secondary care clinicians were excluded. The exact number of potential participants within Australia and New Zealand is unknown given that primary care is delivered through private businesses in these two countries. Best estimates from government and registering organization counts and professional networks of the authors are approximately 31 500 GPs [24], 14 000 nurses [25] and one practice manager per general practice (~ 6900) [24] in Australia; and approximately 4000 GPs, 230 community-based urgent care doctors, 3400 practice nurses [26] and an estimated 900 practice managers in New Zealand.

### Recruitment

A mixture of convenience and snowball sampling was employed for recruitment. Representative groups with large primary care worker memberships were considered most appropriate targets for recruitment to ensure a wide reach and adherence to strict COVID-19 safety requirements. In Australia, potential participants were able to access surveys through a website hosted by the College of Health and Medicine, Australian National University [27]. Organizations including the Royal Australian College of General Practitioners (RACGP), Australian Medical Association and Primary Health Networks disseminated survey links through newsletters. Australian social media groups used by general practitioners and practice nurses also shared study details. Links to the New Zealand survey were disseminated via a number of organizations including the Royal New Zealand Colleges of General Practice and of Urgent Care (RNZCGP and RNZCUC, respectively), General Practice New Zealand, the Rural GP Network, the Practice Managers and Administrators Association New Zealand, primary health organizations, the New Zealand Medical Association and relevant Facebook pages. Implied consent was detailed in participant coversheet, stating that submission of the survey implied consent for use of de-identified data for publication.

All respondents in Australia and New Zealand could sign up to receive email alerts for each new survey. MailChimp was used to automate subsequent survey distribution. Participants were asked to complete each new survey once only, despite the possibility of being exposed to multiple recruitment methods. Multiple methods were used to reduce likelihood of ballot stuffing. Software settings were used to limit to one completion per device. Through a hashing process, each participant’s email address generated a unique alphanumeric token that could not be reverse engineered to identify the participant but could be used to ensure one completion only. IP addresses and postal codes were compared with scan for any inconsistencies in submission origins.

### Data collection tools

Surveys were built, managed and delivered by the United States research team at the Larry A Green Center using SurveyMonkey and MailChimp. Questions were designed jointly by the international research team, which included Australian and New Zealand researchers. Policy-relevant questions were posed by organizational and government representatives with the intention to inform decisions related to the pandemic response. The surveys used a combination of open (qualitative) and closed (quantitative) questions, with data combined and analysed using a mixed methods triangulation design [28]. Over time, surveys used a constructivist paradigm, with participant responses and stakeholder discussions interpreted by the research team to build understanding of the unique problems experienced in primary care, and sequentially inform future survey questions and analysis.

Surveys were designed to be short, taking on average 7 min to complete, and were open for 1 week. All surveys included a series of up to five ‘flash questions’, responsive to the contemporaneous epidemiological and social context, and ‘core questions’. Flash questions were designed to provide timely data on emergent issues in general practice. Topics of these questions were informed by the research team, government policy initiatives (flagged by policy-makers), national news, grey literature [29] and survey responses. In Australia, flash questions were also informed by Primary Health Network representatives (local policy implementers and advocates), and in New Zealand by consultation with general practice leaders. Flash questions were discussed by the research team and prioritized according to immediate policy needs and implications. Questions were adapted to ensure country-specific relevance wherever possible. Due to short turnaround times, piloting of flash questions was restricted to content and face validity and tested by clinical GPs within the research team. Table 1 outlines the timing of data collection and flash question themes. For information on specific questions and response formats, see Additional file 1: Table S2.

Core questions, included in every survey, were based on the survey designed by the Larry A Green Center [19] with adjustments to ensure country-specific language and relevance. For full detail on survey questions and response options, see Additional file 1: Tables S1 and S2. Core questions changed minimally over time, with answer options added relevant to the progression of the pandemic. Topics included perceived practice strain, specific stressors experienced, consultation format (e.g. telehealth or face-to-face), proportion of COVID-19 cases tested and managed, characteristics of participants (doctor, nurse, nurse practitioner, practice manager) and practice descriptors (e.g. billing model, urgent care, urban or rural). New Zealand was unable to collect data identifying Māori practices or region of practice, due to potential for identification. Australia added two survey response options later in the series due to developments of specific practice types for COVID-19. Table 2 outlines the core questions and response formats.

**Table 2:** Core questions and response formats asked repeatedly overtime

Core question	Response format
What is the capacity of your practice to test patients for COVID-19?	4-point Likert scale
Is the current status of COVID-19 putting unusual strain on your practice?	5-point Likert scale
Has COVID-19 led to any of the following stressors in your practice?	Multiple selection
Over the past 2 weeks, how much of the care you provided has been… By video By telephone By e-consultation^A^ In-person Reimbursed	Single-option, grouped proportions of consultations
Over the past 2 weeks, approximately how many suspected COVID-19+ or COVID-19+ people have you (past 8 weeks timeframe used in survey 1) Tested in your practice Triaged and referred for testing Been unable to get tested Treated through your practice Sent to the ED or hospital for treatment monitored at home	Single-option, grouped numbers of patients
Is there anything else you would like us to know about your experience in primary care during this pandemic? Please reflect on both positive and negative experiences	Open text
Is your practice… Owned by GPs Independent and part of a larger group Funded by the State or Territory (Aus only)/District Health Board (NZ only) Owned by a community trust or not for profit A rural practice An Aboriginal Community Controlled Health Organisation (Aus only) Larger than 3 GPs Fully bulk billing (Aus only) Urgent care/afterhours A General Practice Respiratory Clinic (Aus only)^B^ A commonwealth funded vaccine clinic (Aus only)^C^	Binary option, Yes/No
What is your role within your practice?	Multiple selection
What is the postcode of the practice (Aus only)	Open numeric text

^A^Asynchronous tech-based care, provided via a chat function, or text-based app or webpage

^B^Answer option added in series 14 and beyond

^C^Answer option added in series 15 and beyond

### Data management and analysis

Survey data were downloaded and analysed at the end of each survey period. Analysis was completed using SPSS v26 and SAS v9.4 (for Australian data) and Stata 15 (for New Zealand data). Regular data cleaning included creation of new variables to calculate rurality index for Australia on the basis of postcodes using the Australian Bureau of Statistics’ 2017 postcode to 2016 remoteness area concordance. Where a postcode mapped to more than one remoteness category, it was allocated to that with the highest population proportion.

Single survey analysis of quantitative responses included frequencies and percentages to describe each cohort and the primary outcomes of interest, including: practice strain, stressors, consultation format and flash questions. All participants were counted as equal, though reports did include a breakdown of role within the practice [24, 28]. Single survey rapid analysis of open text responses was completed using inductive content analysis by reading, re-reading, developing initial codes and combining codes into general themes. Frequency of codes and relevance to specific flash questions, as well as the overarching research purpose, were used to inform themes. When applicable, triangulated approaches to analysis were employed where quantitative responses were used to support interpretation, inductive coding and thematic development [25]. Themes were reported alongside supportive quotes from participants, and where applicable alongside quantitative results.

In Australia, results were prepared by two researchers within 2 weeks of survey closing. A 2–3 page summary was produced for each survey and checked for accuracy by another two researchers. Each report included a statement on the COVID-19 context in Australia sourced from Australian Government Department of Health data [30], as well as government media releases and news articles. The triangulated mixed methods approach to analysis supported quotes and themes being reported alongside quantitative responses where possible to provide context for identified themes [25]. Summaries were shared with the Department of Health, partner organizations and participants, and published online by the Australian National University [27].

New Zealand results were analysed within 1 week of the survey closing by two researchers. An executive summary and an infographic were created. These were posted on the University of Auckland project website [31]. Executive summaries were emailed to all participating organizations, the Director-General of Health, the Chief Scientific Advisor for the Ministry of Health (who disseminated summaries to key policy personnel in the Ministry) and to the media. Results of the 11 surveys were reported by print, radio or TV media on 21 occasions between June 2020 and February 2021 [32].

Each country merged their own survey data so that participants were treated as cases, and survey questions as variables. Participant responses were matched across surveys within each country on the basis of their survey token ID and/or email address to identify individuals over time (unique respondents). Total participant numbers, number of new respondents and existing participants were calculated for each survey series for each country. Number of surveys completed for each unique respondent was recorded, and a linked longitudinal dataset created using NVivo v12 (QSR International). All participants were counted as equal, though reports include a breakdown of role within the practice. Linking is important to identify individual temporal patterns or possible sources of bias in the qualitative data, affecting future analysis. To describe the total cohort, frequencies and percentages were used to report individual and practice characteristics of unique respondents.

## Results

**Fig. 1:**
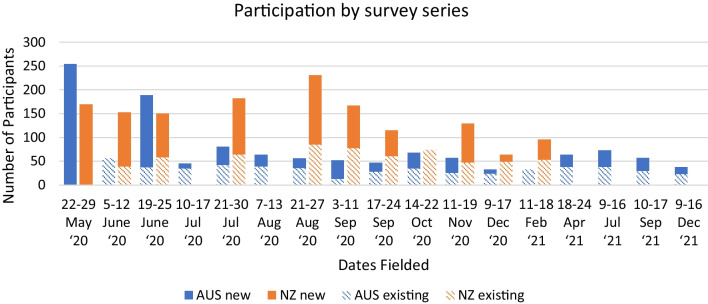
Total new and repeat participant responses for each Australian and New Zealand survey

In Australia, there were 1267 responses to 17 surveys, representing 682 unique participants. In New Zealand, there were 1519 responses to the 11 surveys, representing 482 unique participants. Figure 1 outlines responses to each survey series by country, and the number total participants including new and existing participants.

Response rates were not possible to estimate given convenience and snowball recruitment strategies. Retention varied across surveys. Most participants only completed one survey in the Australia or New Zealand survey series (*n* = 559, 82.0%; and *n* = 291, 60.4%, respectively). Table 3 presents the number of surveys completed by number of unique respondents.

**Table 3:** Number of surveys completed by unique respondents in Australian (17 surveys, *n* = 682) and New Zealand (10 surveys, *n* = 482)

Number of surveys	Australia *n* = 682 *n* (%)	New Zealand *n* = 482 *n* (%)
1	559 (82.0)	291 (60.4)
2	31 (4.6)	67 (13.9)
3	18 (2.6)	23 (4.8)
4	18 (2.6)	26 (5.4)
5	6 (0.9)	28 (5.8)
6	8 (1.2)	18 (3.7)
7	5 (0.7)	11 (2.3)
8	7 (1.0)	6 (1.2)
9	8 (1.2)	6 (1.2)
10	2 (0.3)	3 (0.6)
11	3 (0.4)	3 (0.6)
12+	17 (2.5)	N/A

Most unique respondents were general practitioners. In comparison with Australia, New Zealand had a higher proportion of practice managers (*n* = 21, 3.1% versus *n* = 109, 21.2%) and nurses (*n* = 24, 3.5% versus *n* = 56, 10.9%; respectively). Table 4 presents participant and practice characteristics for each unique respondent.

**Table 4:** Participant and practice characteristics for unique respondents from Australia (*n* = 682) and New Zealand (*n* = 482)

	Australia *n* = 682 *n* (%)	New Zealand *n* = 482 *n* (%)
Participant role description^A^		
GP and practice owner	200 (29.3)	285 (55.6)
GP (non-owner)	460 (67.4)	52 (10.1)
Practice manager	21 (3.1)	109 (21.2)
Practice nurse or nurse practitioner	24 (3.5)	56 (10.9)
Practice description^A^		
GP owned and operated	485 (71.1)	366 (71.3)
Independent and part of a larger group	244 (35.8)	142 (27.7)
State- or territory- (Aus) or District Health Board (NZ)-funded clinic	35 (5.1)	43 (8.4)
Owned by community trust or not-for-profit	40 (5.9)	65 (12.7)
Rural practice	158 (23.2)	104 (20.3)
An Aboriginal Community controlled health organization	21 (3.1)	N/A
Larger than three GPs	583 (85.5)	346 (67.4)
A fully bulk billing practice	149 (21.8)	N/A
An afterhours practice (Aus) or urgent care centre (NZ)	78 (11.4)	84 (16.4)
Commonwealth-funded vaccination clinic^B^	22 (3.2)	N/A
GP-led respiratory clinic^C^	8 (1.2)	N/A

^A^Responses are not mutually exclusive and therefore percentages add to > 100

^B^Answer option added in series 15 and beyond

^C^Answer option added in series 14 and beyond

For both Australia and New Zealand, most unique respondents were from practices with four or more GPs (*n* = 583, 85.5%; and *n* = 346, 67.4%, respectively) and GP-owned and operated practices (*n* = 485, 71.1%; and 366, 71.3%, respectively). Approximately one fifth of respondents from Australia and New Zealand were from rural practices (*n* = 158, 23.2%; and *n* = 104, 20.3%, respectively). Few unique respondents reported being from a state- or territory-funded clinic (*n* = 35, 5.1%) or District Health Board-funded clinic (*n* = 43, 8.4%). While the general cohort is not statistically representative of general practice in Australia or New Zealand, the data are reflective of the range of practice characteristics for both countries, indicating the broad range of primary care voices being represented.

**Fig. 2:**
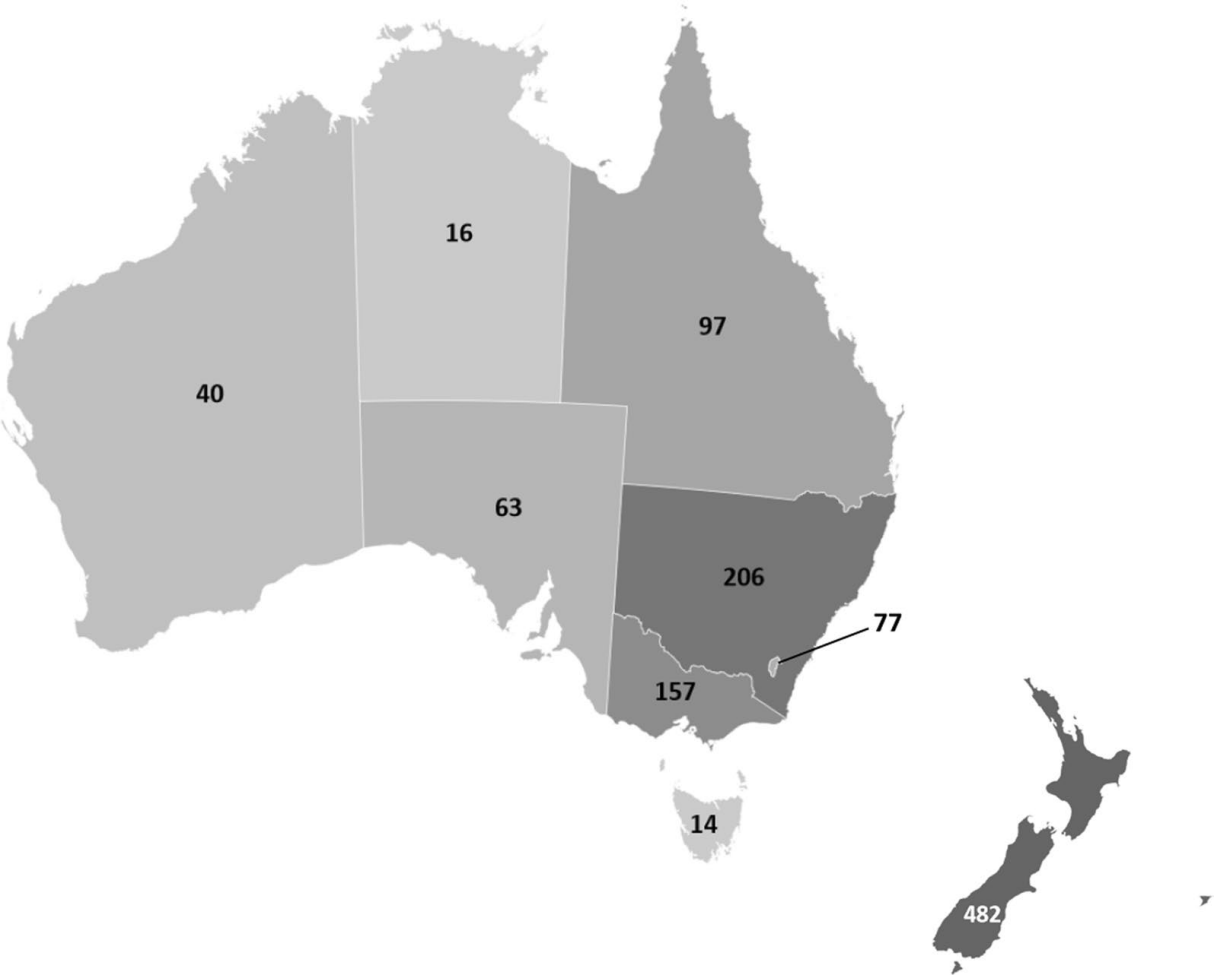
Unique respondents by region of Australia (*n* = 682) and New Zealand (*n* = 482)

Figure 2 shows unique respondents by region. In Australia, unique respondents were obtained from all states and territories, with New South Wales and Victoria, the two largest jurisdictions, contributing the greatest number of respondents (*n* = 198, 29.7%; and *n* = 154, 23.9%, respectively). A total of 12 Australian respondents did not provide data for region. Due to potential identification of participants, New Zealand was unable to collect data on region.

Compared to 2019/20 workforce statistics, respondents in this study were representative of GP distribution around Australia, apart from an overrepresentation from the Australian Capital Territory (11.2% versus 2.0%; *χ*^2^ = 380.01, *p* < 0.01), where the authors are based.

**Fig. 3:**
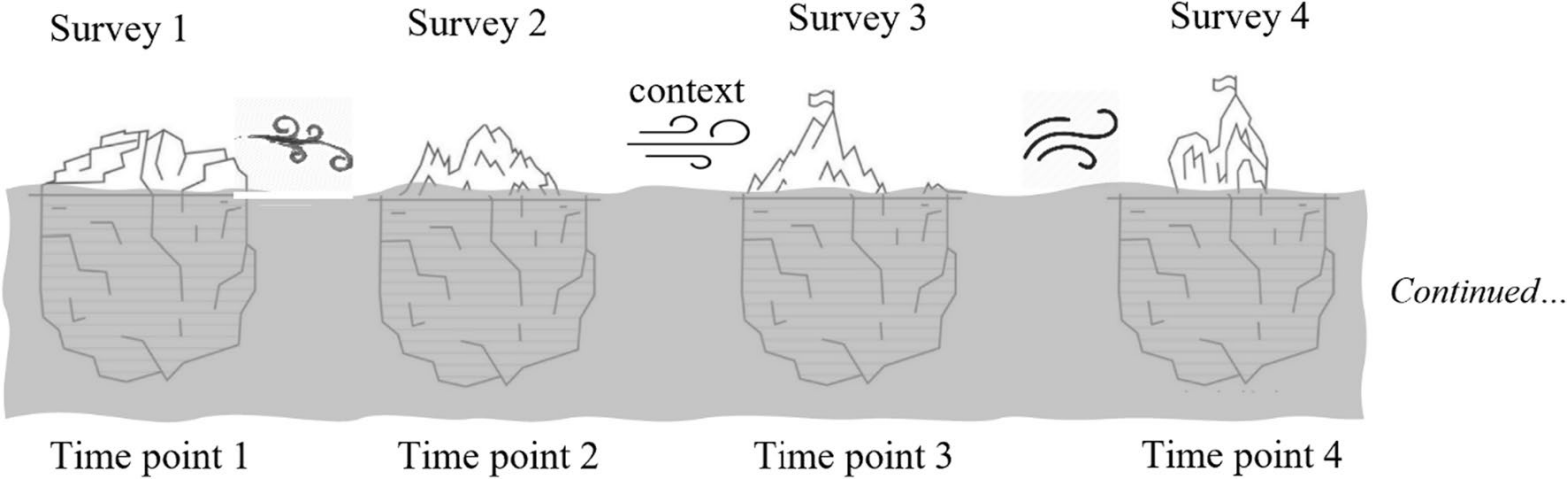
Conceptual model for recurrent cross-sectional analysis, depicting icebergs changing due to environmental forces

To best interpret and understand the data from this study, we created a conceptual analytical design that considers each single survey as an individual dataset (results previously published) and considers the holistic dataset to inform how general practice has responded to COVID-19 over time. Our analytical design used an evolving iceberg metaphor (illustrated in Fig. 3); a conceptualization that addresses many of the weakness of rapid cycle survey methodologies by highlighting the sequential and relatively constructed nature of what is knowable under extreme and changeable conditions. Collectively, responses from participants at any particular timepoint can be considered as a body of information represented by the ‘hummock’ of a given iceberg. The hummock (section of the iceberg exposed above the water) changes over time and space in response to its environment or context. The hummock alone may provide little information about the shape and size of the whole iceberg, especially of the ‘bummock’ (the portion not visible below the waterline). However, observation of changes in the hummock can help illuminate the forces to which the iceberg is exposed, and their progressive impact on its changing form.

## Discussion

### Overview

This pragmatic study design employed responsive, rapid-cycle surveys to inform stakeholders and policy decision-makers about the experience of primary care workers throughout the COVID-19 pandemic in Australia and New Zealand. These methods, incorporating rapid survey development, fielding, analysis, dissemination of results and iterative development that reflected the realities of clinicians, allowed close to real-time communication between research and policy. It also enabled the voice of primary care practitioners to be heard during a period of extreme strain. An emerging challenge of the COVID-19 pandemic has been the need to develop and refine tractable yet robust research methods that can deliver usable results quickly while meeting conventional requirements for rigour and quality, and balancing risks of over-proliferation and wastage amid a “deluge of poor quality research” [33, 34]. While there is a need for flexibility and adaptation of traditional methods, reliability remains an overriding concern [35]. This discussion focusses on an examination of the unique methods employed in this study, and offers a conceptual model for understanding how the methods, their limitations and advantages can be understood and interpreted in the evolving pandemic context.

### Methodological considerations to enhance rigour and interpretation

Pragmatic approaches and a constructivist paradigm to science commonly use novel combinations or adaptations of methodological approaches to apply methods that are best suited to the research question(s), while avoiding philosophical or methodological polarization [21]. Theoretically, pragmatic approaches are concerned with “practical understandings of concrete, real world issues”, and data which can inform the development of actionable knowledge [36]. While the methods used in this study may challenge traditional statistical theory concepts of representativeness; the need for immediate and evolving data to inform decisions, with the dexterity to respond to the changing circumstances of the pandemic, was crucial [15]. The purpose of the project was to describe the experiences of primary care workers during the evolution of the pandemic and to use the findings to give a ‘voice’ to primary care workers by quickly and directly providing the description to policy-makers and representative bodies. As such, the research was exploratory and descriptive, leading to a constructivist view of the unfolding pandemic crisis and its impact in primary care. The concurrent collection of both quantitative and qualitative data supported a triangulated mixed methods analysis with inclusion of quantitative measures to specify and define issues in ways that were simple for respondents to grasp, and which allowed for meaningful interpretation of qualitative responses [28]. The pragmatic approaches used in this study enabled flexibility and responsiveness to a changing environment, adapting to the COVID-19 context, and accepting some of the practical constraints of rapid-cycle research [37, 38]. While not perfect by traditional empirical standards to confirm cause and effect relationships, these methods balance rapidity and rigour to produce evidence that may be ‘good enough’ to inform policy and urgent decision-making while remaining feasible to undertake in critical or heightened operating environments (e.g. a pandemic).

Rapid survey methodologies have been used effectively in public health and field epidemiology [39], though they involve trade-offs between precision and cost. While user-friendly and efficient, rapid surveys may be subject to selection bias, and outputs should be tailored with appropriate statistical and interpretive caution [37]. Despite these constraints, and although single rapid assessments can be limited in their utility and applicability, repeated assessments over time and among different groups can yield important insights [39]. Single survey samples obtained in this study are small and may not be representative, limiting inference and transferability of results. However, over multiple series, this study recruited a wide range of respondents representing a spectrum of practice characteristics, and focussed on generating an illustrative, sequential description of the collective experience of primary care professionals during the evolving COVID-19 pandemic. No other published study currently describes the experience of Australian and New Zealand primary care throughout the pandemic, progressively, as it was experienced. While our individual samples may not capture the entire story at any single interval, as illustrated using the iceberg model, they can iteratively provide information about the changing landscape of the hummock and the environment in which it occurs. As new features emerge, these may warrant investigation, exploration and description to inform future health system and primary-care-specific planning during times of change. Compared with no data, or data that are episodic and disconnected, the value of this method for informing policy decisions is high despite its limitations. Future research may choose to use more traditional and rigorous methods to delve deeper into experiences that were brought to the forefront in this study.

### Strengths and limitations of the applied method

As expected when employing recurrent survey designs, recruitment and retention of participants across the survey series was challenging. The number of responses was higher early in the study, potentially due to the novelty of COVID-19 and the survey, the authority and reach of organizations involved in recruitment, and high motivation to contribute to an understanding of the impacts of COVID-19 in primary care. While surveys developed for this study were short (taking about 7 min to complete), retention of respondents dropped over time with very few participants completing all surveys. Fewer responses over time may have been influenced by sustained high stress and fatigue of frontline primary care workers, and burden of (or reduced interest in) research participation over time. Retention appeared better in New Zealand compared with Australia, perhaps due to stronger media and organizational engagement in New Zealand [32] and lower impact of COVID-19. Of note, neither Australia nor New Zealand have national primary care research infrastructures, such as national practice-based research networks, to support recruitment, data collection or dissemination in primary care, meaning all research participation by primary care workers is wholly in addition to clinical workload, which likely increased participant burden and contributed to fewer responses over time [17, 18]. Limited research infrastructure meant convenience and snowball sampling via national organizations and professional networks was best available for recruitment, though future researchers should note potential restriction of participation to known and engaged parties. The online survey method was preferred during COVID-19 due to strict distancing measures, research ethics restrictions and strong reliance on email and online communication modalities. Still, future researchers should consider paper-based surveys, telephone and/or in person communication to increase responses. Finally, given the short, repeated nature of the survey for immediate feedback, we were unable to collect more contextual data such as primary care worker vaccination opinions or community attitudes towards COVID-19. Where possible, future researchers should triangulate with other datasets, grey literature and published opinion to support the validity of responses.

Limited retention of participants meant longitudinal analysis of individual trajectories within the data was not possible, and alternative approaches are required to describe the composite results of the survey series across time and space. A key strength of this methodological approach is our development of the iceberg metaphor, which provides a model for conceptualizing the relevance and interpretive limitations of our findings. The conceptual model supports integration of qualitative and quantitative data in two ways: data from all participants at a single timepoint can be synthesized and summarized as a unit, then differences and similarities across timepoints can be compared. Crucially however, the changing medical, economic and political context of the COVID-19 environment may have shaped participant responses between surveys. Building on existing rapid feedback reports based on individual surveys [27, 31], and incorporating publicly available government information and media reports, recurrent cross-sectional analysis [40] can be used to tell the story of how COVID-19 and associated policy and public health responses have impacted community-based primary care in Australia and New Zealand over time. Current findings reported elsewhere [24, 28] have highlighted emergent issues, including perceptions on border closures, rural–urban differences in impact [41, 42] and the burden of vaccine counselling experienced in primary care [43].

### Lessons learned

Reflecting on our approach, we offer suggestions for future researchers undertaking similar studies. Early engagement and buy-in from stakeholders was key. Collaboration with influential organizations, such as RACGP and RNZCGP in this study, was essential to assist with recruitment, dissemination of findings and to provide feedback. We established early engagement with policy-makers through established professional networks to provide insight into issues of relevance and to convey findings which may help shape decision-making. Connecting with the media was also important to help disseminate immediate findings and draw the attention of both prospective and previous participants. In this study we placed immediate priority on professional engagement and policy impact with immediate results, while proceeding to academic publication was a secondary and long-term priority. While rapid translation of evidence into practice and policy is becoming more valued in the research world, traditional metrics of publication are still highly regarded [44, 45], and studies employing this method should also plan for longitudinal analysis and dissemination to ensure maximum impact.

## Conclusions

The COVID-19 pandemic has been a time of clinical and social uncertainty and upheaval that has placed a profound burden on primary care professionals. Understanding these changes has required an equally rapid adaptation in research methods. This rapid-cycle, recurrent survey identified and responded to immediate issues experienced in community-based primary care settings, highlighting, probing and communicating these to relevant professions and policy decision-makers in near real-time instalments. As such, the method proves feasible and accessible to implement, particularly during times of rapid change. Future research using a repeated cross-sectional approach should consider applying the conceptual model presented here to provide a rich, longitudinal description of data.

### Supplementary Information

**Additional file 1:** **Table S1 and S2.** Questions and answer formats for all questionnaires in Australia and New Zealand during the study period. List of all question and answer options for all surveys conducted in Australia and New Zealand relating to this study.

## Data Availability

Data generated and used for this paper are not publicly available due to being re-identifiable. De-identified data may be available by reasonable request to the country team leaders: For Australian data, contact Professor Kirsty Douglas (Kirsty.a.douglas@anu.edu.au). For New Zealand data, contact Professor Felicity Good-year Smith (f.goodyear-smith@auckland.ac.nz).
